# A Telemedicine Platform for Aphasia: Protocol for a Development and Usability Study

**DOI:** 10.2196/40603

**Published:** 2022-11-24

**Authors:** Monara Nunes, Ariel Soares Teles, Daniel Farias, Claudia Diniz, Victor Hugo Bastos, Silmar Teixeira

**Affiliations:** 1 Federal University of Piauí Regeneração Brazil; 2 Federal Institute of Maranhão Parnaíba Brazil; 3 Federal University of Delta do Parnaíba Parnaíba Brazil

**Keywords:** aphasia, serious games, deep learning, telemedicine, diagnosis, treatment, language, machine learning, rehabilitation, smart platform

## Abstract

**Background:**

Aphasia is a central disorder of comprehension and expression of language that cannot be attributed to a peripheral sensory deficit or a peripheral motor disorder. The diagnosis and treatment of aphasia are complex. Interventions that facilitate this process can lead to an increase in the number of assisted patients and greater precision in the therapeutic choice by the health professional.

**Objective:**

This paper describes a protocol for a study that aims to implement a computer-based solution (ie, a telemedicine platform) that uses deep learning to classify vocal data from participants with aphasia and to develop serious games to treat aphasia. Additionally, this study aims to evaluate the usability and user experience of the proposed solution.

**Methods:**

Our interactive and smart platform will be developed to provide an alternative option for professionals and their patients with aphasia. We will design 2 serious games for aphasia rehabilitation and a deep learning–driven computational solution to aid diagnosis. A pilot evaluation of usability and user experience will reveal user satisfaction with platform features.

**Results:**

Data collection began in June 2022 and is currently ongoing. Results of system development as well as usability should be published by mid-2023.

**Conclusions:**

This research will contribute to the treatment and diagnosis of aphasia by developing a telemedicine platform based on a co-design process. Therefore, this research will provide an alternative method for health care to patients with aphasia. Additionally, it will guide further studies with the same purpose.

**International Registered Report Identifier (IRRID):**

PRR1-10.2196/40603

## Introduction

Aphasia is a language disorder caused by damage to 1 or more areas of the brain that control some or all language modalities, including the expression and comprehension of speech, reading, writing, and gestures [1]. Although many people have aphasia because of stroke, other sources of brain damage can cause it (eg, head trauma, brain surgery, epileptic disease, and neurodegenerative syndromes) [2-4]. When caused by neurodegenerative syndromes, aphasia can be diagnosed as primary progressive aphasia and classified as logopenic, semantic, and nonfluent/agrammatic [3]. When caused by stroke, it can be classified as anomic, Broca (motor), Wernicke (sensory), global, conduction, and transcortical (motor, sensory, and mixed) [4,5]. As the main impairments of aphasia are related to the expression and comprehension of language, aphasia can be divided into 2 large groups: expressive and receptive [6].

The aphasia diagnosis is usually made by a neurologist or speech therapist, based on the clinical and pathological features to characterize the language disorder [7]. Discriminating the different types of aphasia is a complex task since signs such as impaired speech comprehension and/or articulation, inability to repeat, impaired semantics, and difficulty in naming objects are present in various types of aphasia, whether due to neurodegenerative disease or by the interruption of cerebral flow [8]. To make the diagnosis of aphasia simpler and more precise, computational solutions have been developed to classify/detect the types of aphasia. The proposed computational techniques have used classical machine learning [9-13], deep learning (DL) [14,15], and fuzzy logic [16,17]. DL techniques have shown better results in different performance metrics to classify aphasia [14,15].

Once the patient is diagnosed, the treatment of aphasia is a challenge for health care professionals. Patients with expressive aphasia appear to be the easiest to rehabilitate, because these patients can understand the method used in a particular rehabilitation technique or, at least, the instructions for using it. However, there has been little success in receptive aphasia rehabilitation trials [6,18]. Serious games have shown promise in the rehabilitation of diseases of neurological causes, as they can lead to engagement and offer feedback to the patient and therapist on the progress of therapy [19,20]. To the best of our knowledge, few studies have focused on serious games for aphasia rehabilitation [21,22]. In such studies, therapy is only suitable for patients with a good understanding of the task, thus neglecting patients with receptive aphasia. Unlike previous studies, our work will focus on both the development of a platform to aid in the aphasia diagnosis using DL techniques and the application of serious games suitable for patients with receptive and expressive aphasia.

This paper presents a protocol of a study that has 2 objectives: (1) to implement a computer-based solution (ie, a telemedicine platform) that uses DL algorithms to classify aphasia and, following a co-design study, to develop 2 serious games for the treatment of aphasia; and (2) to evaluate the usability and user experience of the developed games.

## Methods

### Study Design

**Figure 1:**
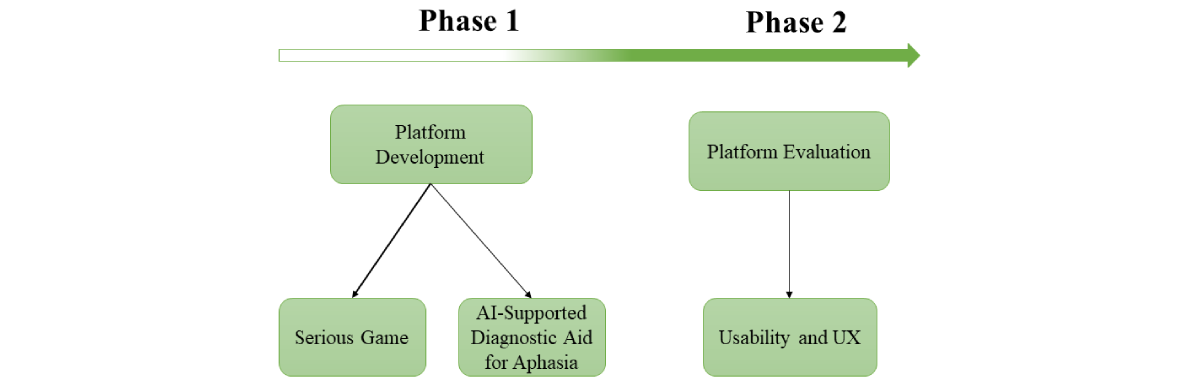
Flowchart of the study phases. AI: artificial intelligence; UX: user experience.

The study will be divided into 2 phases, as can be seen in Figure 1. The first phase refers to the implementation of a platform to aid in the diagnosis and treatment of patients with aphasia. The second phase refers to a study to evaluate the implemented serious games for aphasia rehabilitation. It will focus on the evaluation of usability and user experience by patients with aphasia and speech therapists.

### Phase 1: Platform Development

#### Literature Search and Questionnaire

A literature search was carried out in June 2022 to identify content related to aphasia rehabilitation using serious games and content related to aphasia classification using computational solutions. This literature search was carried out in the main digital libraries of the medical field, including PubMed and Web of Science. We developed the first version of the game content based on studies [21,22] that used virtual reality tasks with oral expression, articulation, listening comprehension, cognition, articulation, semantics, naming, and repetition. Furthermore, these studies reported on the attractiveness and satisfaction when users are playing a serious game to rehabilitate aphasia. In the studies using computer-based solutions to classify aphasia [14,15], techniques of machine learning and DL have been applied. With these works, we have learned about the best classification techniques and audio features to classify aphasia.

In parallel with the literature search, a questionnaire with 12 open and closed questions that address the clinical needs for the diagnosis and treatment of patients with aphasia was directed to a group of 10 speech therapists with more than 1 year of experience in aphasia rehabilitation. This was a convenience sample, and the recruitment was carried out via social networks. From this questionnaire, we identified that 60% (n=6) of participants reported performing the aphasia diagnostic before starting treatment, and 50% (n=5) used a device to perform this diagnosis. When investigating the difficulties that speech therapists have when performing the diagnosis, the sample reported the lack of resources for rapid testing, standardized protocol, diversity of symptoms of each aphasic syndrome, and absence of imaging tests and family assistance. As mandatory items for the diagnosis, 80% (n=8) of the participants reported that speech comprehension, object naming, and sentence repetition are relevant characteristics for diagnosis; 70% (n=7) reported speech articulation as relevant characteristic; and 10% (n=1) reported speech expression, lesion size, and underlying disease. For aphasia rehabilitation, 90% (n=9) reported that semantic tasks are essential; 80% (n=8) reported speech articulation and naming tasks as essential; 70% (n=7) reported repetition tasks; and 10% (n=1) reported tasks of cognitive function and socioeconomic impacts.

From the results of the questionnaire and the literature search, we developed content that will be presented to a group of 3 speech therapists, 2 software developers, and 2 neuroscientists to receive feedback. This will enable us to improve the content. The sample is consistent with researchers who accepted being part of the study development process, and the form of recruitment was by invitation.

This group will meet weekly for 1 hour. At this time, a 5-minute video that includes updated content will be presented, and participants will be encouraged to rate the product from 0 to 10, with 0 corresponding to the worst rating and 10 an excellent rating. In addition, participants will be encouraged to justify their rating and suggest improvements to the proposed solution. During all meetings, improvements reported by the group will be enumerated and categorized for later implementation.

#### Serious Game Design

Aphasia may be divided into 2 major groups: expressive aphasia and receptive aphasia. Each type has characteristics and, therefore, different therapeutic methodologies. By considering such groups, 2 games will be developed.

#### Game 1: Expressive Aphasia

The objective of this game is to stimulate tasks for expressive aphasia, such as articulation, semantics, naming, repetition, prosody, and emotional tone in utterances. Based on the current literature and discussions with the research team, the initial proposal has already been designed, which is described hereafter.

The game’s plot is inspired by a treasure hunt, where the patient will need to open as many chests as possible and collect the treasures in a time interval determined by the therapist. After the set interval runs out, a new game environment will be unlocked, and the user will be able to collect more treasures. To open each chest and collect the treasure, the user must perform a specific task for each environment. The therapist will be assigned the role of deciding which environments the patient should go through. The number of opened chests and collected treasures will not be decisive for the user to move from one environment to another. This rule was defined so that users go through all types of environments defined by the therapist, regardless of their level of language impairment. The more the user collects treasures in each environment, the greater the complexity of the task in that environment will be. The complexity of tasks within each environment will be based on the following 4 elements:

1. Sentence size: This element refers to the number of words to build a sentence. Sentences with 3 to 4 words will correspond to the basic level; 5 to 6 words will correspond to the intermediate level; and more than 6 words will correspond to the advanced level.
2. Number of clauses: This element refers to the number of verbs present in the sentence. Sentences with more than 2 verbs are considered complex. The presence of 1 verb will correspond to the basic level; the presence of 2 verbs will correspond to the intermediate level; and the presence of 3 or more verbs will correspond to the advanced level.
3. Presence of adverbs: An adverb is a word that indicates a circumstance (mode, time, or place). It can modify a verb, an adjective, or another adverb. The presence of adverbs as well as adverbial phrases in the sentence will be indicative of more complex sentences. Adverbs will only be present at the intermediate and advanced levels. At the intermediate level, there will be only 1 adverb, and at the advanced level, there will be 2 or more adverbs.
4. Number of syllables: This element refers to the number of syllables to compose a word. One syllable will correspond to the basic level; 2 syllables will correspond to the intermediate level; and 3 syllables will correspond to the advanced level.

To give feedback on the number of chests that still need to be opened in the environment, a map with the location of the chests and the user will be fixed on the right side, in the lower corner of the screen. In all, there will be a total of 4 environments, each one being designated to a type of task: articulation, semantics, naming, and repetition of sentences. Each environment will have a total of 10 chests to open. The user will be allowed to save the game or pause it without any penalty.

#### Game 2: Receptive Aphasia

Similar to the previous game, the game proposal was based on the current literature and discussions with the research team and will be described hereafter. We will discuss it with speech therapists, scientists experienced in serious game development, and the development team. After approval, the proposal will be implemented.

The initial proposal of the game’s plot is inspired by activities of daily living, where the user must complete some missions that he or she comes across daily, such as entering the elevator and pressing the button to go to the next floor, buying bread at a bakery, or taking out the garbage. The purpose of the game is to work the patient’s cognitive skills so that he or she becomes as functional as possible using nonverbal language to complete the missions. The therapist will be able to choose which mission the patient should undertake and the time for the completion of the mission. If the patient is unable to complete the mission in the time set by the therapist, hints with arrows indicating the direction will appear to guide the patient in the mission. After being completed, the mission will be unlocked in case the patient wants to try again without the tips.

A total of 10 missions with different contexts will be implemented. Finally, players will be allowed to save the game or pause it without any penalty.

#### Tools Used for the Development of the Serious Game’s Platform

The games will be developed over a virtual reality platform, as it increases the immersion in the game and the effectiveness of the intervention. In this way, the number of participants for aphasia treatment can be increased. User interaction with the game environment will be performed using 2 options: (1) gaze, with interaction using eye movements; and (2) head movements. These options will add a mouse-like interaction, which is an on-screen pointer that users will be able to move.

For the development of the platform, the *AFRAME* library made in Javascript was selected to create a virtual reality environment. Since no additional framework was used alongside *AFRAME*, the *Parcel* Javascript compiler was used to allow the use of modern Javascript features. Finally, the project will be hosted on the Vercel cloud platform (Vercel, Inc).

### Diagnostic Model Development

#### DL Algorithms and Model Training

At this stage of the research project, we went through a process of developing a model for classifying aphasia. For the development of this model, we will use the DL technique since, according to previous studies, it presents better results to classify aphasia through acoustic data [14,15]. In all, 3 DL algorithms will be used, namely: LeNet, Resnet-34, and SqueezeNet. To acquire data for training the model, vocal data will be collected from patients with aphasia diagnosed by a neurologist or speech therapist and individuals without aphasia whose ages are matched to the aphasia group (see details in Data Set Creation section). The collection of vocal data is made through a collection app developed to record the voices from patients during specific tasks. The app development as well as data collection tasks are described in the following sections. After data acquisition, we will conduct a data preprocessing step. In this step, noise and bad quality audio that may impair the performance of the models will be removed. The extraction of features in DL is an automatic process, where it is not possible to know which features were chosen for training the model. After training the models, an evaluation will be performed using performance metrics derived from the confusion matrix (accuracy, sensitivity, specificity, *F*_1_-score, receiver operating characteristic curve, and area under the curve). The best performing model will be deployed to the platform.

#### Development of the Data Collection Application

A hybrid mobile app was developed with Ionic (graphics component library), VueJS 3.0 framework, and Capacitor as a native functionality library. The app was written in Javascript, specifically using the *Typescript* superset. The system allows users to manage the participant data (adding, editing, and deleting), as well as collecting audio within a defined protocol for aphasia tracking. The app can be installed via an .apk file or used through the browser. The collection consists of the audio recording of the participants for an indefinite period and an infinite number of times. All files are sent to Dropbox, which is a cloud storage service, for further analysis and discussion.

#### Data Set Creation

### Consent to Participate

Participants will be informed about the purpose of the research, its objectives, and procedures. They will be consulted regarding their acceptance to participate in the study. After clarification, the participants or their guardians will be instructed to sign the Free and Informed Consent Term approved by the Ethics and Research Committee of the Federal University of Piauí, guaranteeing anonymity and freedom of absence from the research, as well as the realization of clarification regarding the same and the right to withdraw from their participation during the study.

### Study Participants

The inclusion criteria for participants include providing a signed consent form, agreeing to the study procedures, being available for the study duration, and being aged 40-80 years. For the aphasia group, participants must have a diagnosis of aphasia after stroke given by a specialized health professional, with a post-illness time of 6 months. Regarding the exclusion criteria, patients who do not present a diagnosis and classification of the type of aphasia or who have severe cognitive impairment (Mini-Mental State Examination score less than 22 points) and severe aphasia were excluded. For the control group, participants must be healthy and without a history of language or cognitive disorders.

### Recruitment

Participants with aphasia will be recruited at the physiotherapy school clinic of the Federal University of Delta do Parnaíba, Parnaíba; at the Dirceu Arcoverde State Hospital, Parnaíba; and at the Neurology Institute Deolindo Couto linked to the Federal University of Rio de Janeiro, Rio de Janeiro, Brazil. Healthy participants will be recruited from advertisements in the cities of Parnaíba and Rio de Janeiro, Brazil. The recruitment and screening of participants will be performed by a trained speech therapist. At this moment, the participant’s cognitive level, possible language impairments, and aphasia level will be investigated. As this is a sample group for training a DL model, the sample size will be time-oriented, in which the largest number of individuals will be recruited within 6 months.

### Vocal Data Collection

**Figure 2:**
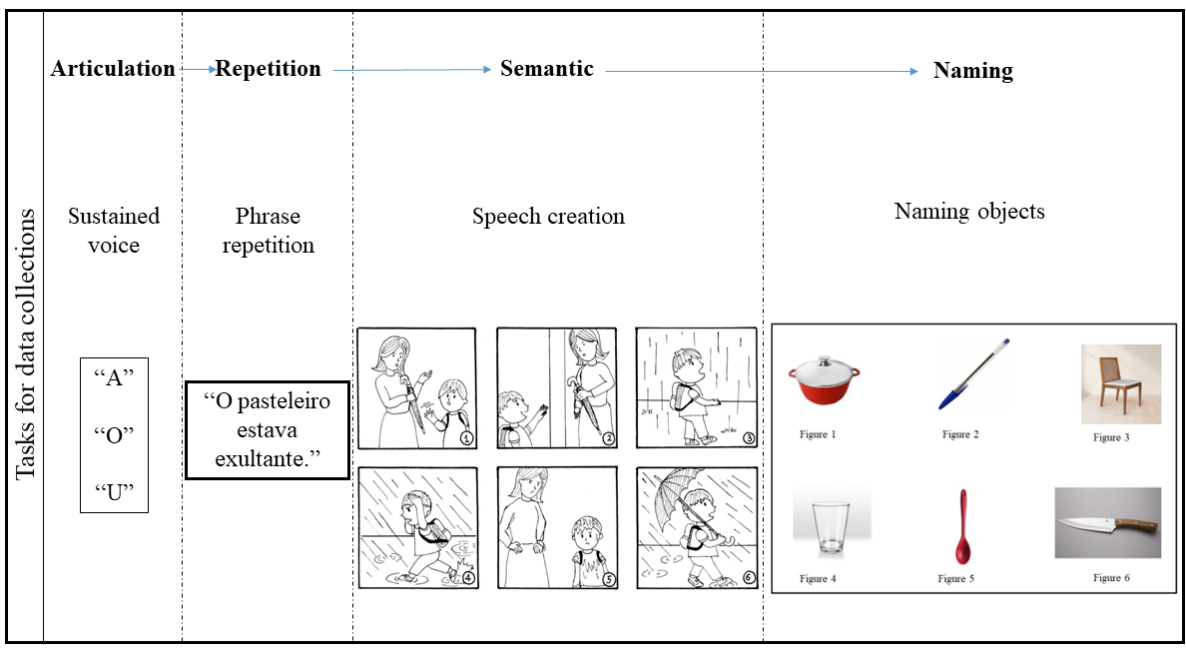
Scheme of tasks used for data collection.

Data will be collected from audio samples from participants with aphasia or healthy participants. Therefore, for this study, participants will be placed in 2 conditions: with aphasia and control (without aphasia). Data collection will be conducted in a noise-free environment through a mobile app for voice capture. The app presents tasks where the participant must sustainably repeat the vowels “a,” “o,” and “u” and the predefined phrase: “O pasteleiro estava exultante” (“The pastry chef was overjoyed” in Portuguese). These commands are reported in the literature to discriminate against the type of aphasia, since it allows the assessment of speech comprehension and articulation [23]. In addition, figures are presented to the participants, and they will be instructed to name them or develop a speech about the figures (see Figure 2). Those images were taken from the Talkbank database, which are widely used to assess speech and gesture variations in patients with aphasia [24].

#### Ethics Approval

The study was approved by the Ethics Committee of the Federal University of Piauí (5.134.321) in October 2021.

#### Phase 2: Usability Study

##### Platform Schedule

Participants, on a first visit, will receive training on how to use the platform. Speech therapists will be encouraged to customize a different treatment for 2 patients with aphasia, which results in 2 treatments per speech therapist. Patients with aphasia will be encouraged to use the platform for 30 minutes, 3 times a week, for 2 consecutive weeks. The task to be performed will be customized by the speech therapist. After the 2-week period, all participants will be encouraged to complete a usability questionnaire as accurately as possible.

##### Usability Study Participants

Inclusion criteria for participants include (1) speech therapist with at least 1 year of experience in treating patients with aphasia; and (2) patients with a diagnosis of aphasia after stroke given by a specialized health professional, with a post-illness time of 6 months, and a Mini-Mental State Examination score more than 22 points. All participants must provide a signed consent form, agree to the study procedures, and be available for the study duration. Participants who do not complete the study protocol will be excluded.

##### Tool to Test the Usability of the Platform

**Figure 3:**
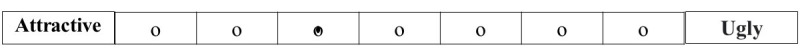
Example of a User Experience Questionnaire question.

To assess the usability of the computer system, the User Experience Questionnaire (UEQ) will be applied, consisting of 26 items, including 6 factors: Attractiveness, Perspicuity, Efficiency, Reliability, Stimulation, and Novelty. The questionnaire consists of pairs of opposites related to the properties that the product has [25]. The graduations between opposites are represented by circles. By marking one of the circles, the user expresses his or her opinion about a concept. See Figure 3 for an example of a UEQ question.

The user will be asked to mark their answer spontaneously, being instructed not to think too much about the answer. The user will also be asked to always choose an answer even if they are unsure about a couple of terms or the terms do not fit the product as there are no “right” or “wrong” answers—opinion is what counts.

##### UEQ Statistical Analysis

The data analysis will consist primarily of descriptive statistics, and outcomes will be described primarily in percentages or proportions. Data will be analyzed using SPSS statistical software (version 25; IBM Corp). Microsoft Excel (version 16.1) will be used for charts. Participants’ responses will be independently analyzed by 2 researchers, and data will be entered twice to minimize typing errors. After analysis by researchers, the consensus among the experts will be evaluated using the Cohen κ coefficient. Statistical significance will be considered at a 2-tailed *P* value of <.05.

## Results

Data collection began in June 2022 and is currently ongoing. Results of system development as well as usability should be published by mid-2023.

## Discussion

### Hypothesis and Significance

We hypothesized that a platform that makes use of DL algorithms will be able to classify aphasia from a set of vocal data and that only this data will be sufficient to classify aphasia. In addition, it is accredited that a platform that offers web-based tasks in the format of serious games will be well evaluated by speech therapists and patients with aphasia who make use of it.

It is expected to provide an alternative that facilitates the diagnosis of aphasia and allows a treatment directed to the type of impairment of the patient with aphasia. Speech therapists assume the role of the prescriber of the treatment and follow-up of the patient. The therapists will be involved from the elaboration of the treatment to the finalization of the process of creation and analysis of the platform. Patients and caregivers are assigned the role of evaluator and system user.

DL techniques, which are applications of artificial intelligence, have recently emerged and are now rigorously applied in the medical field. DL refers to the use of artificial neural networks with multiple hidden layers [26]. The use of DL with vocal data to classify aphasia has been the objective of studies, both to classify the type of aphasia [14] and to estimate severity [15]. Such studies suggest that DL models using vocal data can estimate aphasia in patients with early-stage acute stroke. These findings justify further research to assess the applicability of DL models in different study populations.

Rehabilitation technologies have the potential to increase the intensity and dose of rehabilitation, improve access to rehabilitation, reduce therapists’ workload, measure and provide feedback on performance and recovery, and engage and motivate patients [27]. Thus, serious game systems based on virtual reality have become popular in medical rehabilitation and can be used as a new alternative therapy method for language recovery in patients with aphasia [21,22]. Serious games to be proposed will be intended for patients with expressive and receptive aphasia. Therapeutic approaches are aimed at cognitive and expressive speech recovery, which are fundamental in the rehabilitation process of such patients.

### Expected Clinical Impacts

The study proposed here will bring clinical, scientific, and socioeconomic impacts as it aims to serve as a diagnostic aid and alternative for the treatment of patients with aphasia within their clinical particularities. It is expected that the use of DL will be promising in the aphasia classification process and thus facilitating the diagnosis and clinical decision. Once classified, the therapist’s conduct must be directed to the patient’s needs. Here, we propose telerehabilitation as a way to reach a larger audience, in addition to reducing long-term disability, increasing secondary prevention, and allowing follow-up after the acute phase of treatment, thus increasing responses to therapy and encouraging continuity of care. Serious games have been gaining public attention and are becoming an object of interest to researchers, as they can be introduced in various fields of medical practice, since they imply greater patient engagement in therapy [27]. It is hoped that the games to be developed from this proposed study will make patients more enthusiastic and more willing to talk, especially when exposed to virtual reality technology that will provide the creation of interactive worlds in which the patient can experience new therapeutic approaches that would not be accessible in the real world. In addition, with the platform improvement, it is expected that populations that would not possibly receive continued care can have support, thus configuring a socioeconomic impact.

### Limitations

The sample sizes used in building the training and testing data sets of DL models in aphasia classification studies may be relatively small. However, our goal will be to capture a sample size that allows the training and testing of DL models with acceptable results in different performance metrics. The collection time will also influence the participant's permanence in the study, which will also be influenced by the availability of those involved. A system usability study does not need a large sample number, and therefore, usability results may not be generalizable and may be specific to the community and environment. However, this study may serve as a basis for others, and new studies with the tool proposed here should be developed to prove its effectiveness.

### Conclusions

The expected results of this study will provide a basis for a successful technological development study. The results will produce an appropriate intervention for patients with aphasia and indicate challenges and opportunities for the refinement of study procedures, including methodology for collecting data from patients with aphasia, creating serious games, ways to assess user experience, and using DL to classify vocal samples.

## Abbreviations

DL: deep learning
UEQ: User Experience Questionnaire

## Data Availability

Data sharing not applicable to this paper as no data sets were generated or analyzed during the current study.
